# Contraceptive method choice by type of intimate partner violence: findings from the 2015 Colombian demographic and health survey

**DOI:** 10.1186/s12978-026-02478-8

**Published:** 2026-10-24

**Authors:** Emma Sophie du Marchie Sarvaas, Signe Svallfors, Mariano Salazar

**Affiliations:** 1 Department of Global Public Health, Karolinska Institutet, Tomtebodavägen 18a, Widerströmska Huset, Stockholm, 171 77 Sweden; 2 Department of Sociology, Stockholm University, Universitetsvägen 10B, Stockholm, 10691 Sweden

**Keywords:** Intimate partner violence, Contraception, Reproductive autonomy, Colombia

## Abstract

**Background:**

Intimate partner violence (IPV) is a major global health issue and limits women’s reproductive autonomy. Despite high IPV and contraception prevalences in Colombia, the link between specific types of IPV and contraceptive methods remains understudied. This study aimed to examine associations between IPV and the type of contraceptive method used by Colombian women.

**Methods:**

This cross-sectional study used data from the 2015 Colombian Demographic and Health Surveys. The sample included 13,896 ever-partnered women aged 15–49 who were not pregnant, infecund, or used permanent methods. Contraceptive method was categorised as: no contraception, traditional/fertility awareness methods (FAM), intrauterine devices (IUDs)/implants, oral/injectables, and external condoms. Exposure variables included any IPV, physical, sexual and emotional violence, and controlling behaviour in the past 12 months. Adjusted multinomial logistic regressions were conducted, with a significance level of *p* < 0.05.

**Results:**

Physical and emotional IPV were associated with higher relative risks of using traditional/FAMs and IUDs/implants, and lower probabilities of using oral/injectables or external condoms. Sexual IPV was associated with higher relative risk and controlling behaviour with lower relative risk of using all contraceptive methods.

**Conclusions:**

IPV-exposed women were more likely to use long-acting methods that can be used covertly, are less susceptible to partner interference, and require fewer healthcare visits, which may reflect increased contraceptive vigilance. Understanding the impact of IPV on contraceptive methods is necessary to improve women’s reproductive autonomy. Integrating IPV screening into family planning services and further research into contraceptive decision-making in abusive relationships is recommended.

**Supplementary Information:**

The online version contains supplementary material available at https://doi.org/10.1186/s12978-026-02478-8.

## Abstract (plain language summary)

Intimate partner violence (IPV) is a common problem that can affect women’s health and wellbeing, including their ability to make decisions about contraception. Women who experience IPV may face pressure, control, or fear that limits their reproductive choices. However, little is known about how different forms of IPV influence the type of contraception women use.

This study examined the relationship between IPV and contraceptive method choice among women in Colombia. We analyzed data from a large national survey of women aged 15–49 years who had ever been in a relationship. We looked at several forms of violence, including physical, sexual, and emotional violence, as well as controlling behaviors by a partner.

We found that women who experienced IPV were more likely to use some contraceptive methods than others. In particular, women exposed to physical, emotional, or sexual violence were more likely to use long-acting methods such as intrauterine devices (IUDs) and implants. These methods can be used without a partner’s knowledge and are less vulnerable to interference. Women who experienced controlling behavior from a partner were generally less likely to use contraception.

Our findings suggest that women may adapt their contraceptive choices in response to violence and control within partner relationships. Family planning services should consider the impact of IPV on women’s reproductive decisions. Integrating support and screening for violence into contraceptive services may help women access methods that best meet their needs and strengthen their reproductive autonomy.

**Supplementary Information:**

The online version contains supplementary material available at https://doi.org/10.1186/s12978-026-02478-8.

## Background

An estimated one in three women worldwide has, at some point in her life, been victim of physical or sexual violence by a current or former male intimate partner [1]. The World Health Organization (WHO) defines intimate partner violence (IPV) as “*the behaviour within an intimate relationship that causes physical*,* sexual or psychological harm*,* including acts of physical aggression*,* sexual coercion*,* psychological abuse and controlling behaviours. Both by current and former spouses and partners*” [1].

IPV is considered a major global health issue and a violation of women’s human rights with studies linking it with women’s poor physical, mental, sexual and reproductive health [2]). Thus, the elimination of all forms of violence against women, including IPV, is included in the Sustainable Development Goals (SDGs) target 5.2 [1]. Exposure to IPV has been also shown to undermine women’s ability to make informed and independent decisions about their reproductive health, such as using contraception, becoming pregnant, or continuing a pregnancy, commonly referred to as reproductive autonomy [3–5]. Moreover, studies have shown that women who have experienced IPV have a higher risk of unintended pregnancies, miscarriages, stillbirths, preterm labour, and contracting sexually transmitted infections (STIs), among other adverse outcomes [3–5].

Contraception is central to reproductive autonomy, allowing women to delay, space or prevent pregnancies, and prevent STIs. Globally, modern contraceptive use has increased to 43·9% among women aged 15–49 in 2025, with regional variations from 27·0% in Africa to 55·2% in Latin America and the Caribbean [6]. Female sterilisation and intrauterine devices (IUDs) are the most common methods globally [6]. Modern contraceptive use has been associated with socioeconomic and cultural factors at the individual, community, and health system level [7]. Individual factors associated with lower contraceptive use include IPV, poverty, low educational attainment, relationship status, and a lack of knowledge about contraception and their side effects [7]. At the community and societal level, attitudes among women’s partners, families and peers toward contraception can facilitate or obstruct contraceptive use [7]. Finally, health system characteristics such as contraception accessibility, availability, and staff training (or lack thereof) can also influence women’s contraceptive use [7].

At the individual level, women’s exposure to different forms of IPV has been associated with both increased and decreased contraceptive use [5, 8–17]. These seemingly conflicting findings might be related to methodological variations in how IPV and contraceptive use have been measured, sample characteristics, and the pathways by which violence influence contraception. For example, studies around the world have found that different forms of IPV were associated with reduced condoms and oral contraceptives use [8, 9, 12], which might be related to power imbalances within the couple that limit women’s ability to access, negotiate, or continue contraceptive use [10, 11]. On the other hand, studies have reported increased contraceptive use among women exposed to IPV [13–15], such as IUDs and implants [18], which may represent a strategy to avoid dependence on an abusive partner or to prevent pregnancy out of concern that their children could also be affected by violence.

In Latin America, very few studies have evaluated the association between contraceptive use and IPV exposure. Population-based studies in Nicaragua [5] and Honduras [16] reported that women exposed to any IPV form [5], physical IPV [5] or emotional IPV [16] had higher odds of using contraceptives than the unexposed. A study conducted in Colombia among young women aged 13–24 years reported a positive, though non-significant, association between sexual IPV and contraceptive use [19]. Overall, while these studies above provided valuable data, they also have methodological limitations, including assessing only overall contraceptive use without differentiating between specific methods [5, 16, 19] examining only certain forms of IPV such as physical or sexual IPV [5, 16, 19], focusing on lifetime IPV exposure rather than IPV experienced within the past 12 months [16, 19], and focusing on only young adults [19]. These limitations preclude policymakers from distinguishing how specific forms of IPV are associated with the use of different contraceptive methods, which may be key to understanding the contradictory findings reported in previous studies.

In this paper, we aim to address this research gap by evaluating the associations between physical, sexual, emotional IPV, and controlling behavioural in the past 12 months and the type of reversible contraceptive method used by ever-partnered Colombian women aged 15–49 years who participated in the 2015 Demographic and Health Surveys (DHS). We used data from the 2015, which was the most recent nationally representative ENDS dataset available when the study was conducted in 2024. The subsequent survey round, originally planned for 2020, was postponed due to the COVID-19 pandemic and was conducted during 2024–2025 [20]. We focus on IPV in the past 12 months as it may more accurately reflect the current context in which reproductive decisions are made. We hypothesise that IPV-exposed women are less inclined to use partner-dependent methods (e.g., external condoms) and more prone to using methods that require less frequent contacts with the healthcare system and may be less susceptible to partner interference (e.g., IUDs and implants).

## Methods

This study was set in Colombia, an upper-middle income country in South America with remarkable family planning progress since the 1960s [21]. Modern contraceptive use prevalence reached 60% in 2025. Female sterilisation was the most common, followed by injectables, and oral contraceptive pills (OCPs) [21]. Data from the 2015 Colombian DHS showed that 64.0% of women had ever experienced psychological IPV (including controlling behaviours), 31.9% physical IPV, 7.6% sexual IPV, and 57.9% controlling behaviours [22]. In contrast, the 2025 Colombian DHS reported substantially lower lifetime prevalence estimates among women aged 13–49 with a history of union: 17.7% for psychological IPV, 7.9% for physical IPV, and 2.0% for sexual IPV. In the 2025 survey, psychological IPV included controlling behaviours, of which jealousy or anger when a woman spoke with another person was the most frequently reported (13.9%) [20]. However, direct comparisons between the 2015 and 2025 estimates should be made with caution because of changes in the measurement instruments and definitions used to assess IPV across the two surveys [20].

The DHS divides Colombia into six geographical regions (Atlántica/Caribe, Oriental, Central, Pacífica, Bogotá, and Orinoquía–Amazonía), which differ substantially in their socioeconomic and ethnic composition [23]. Multidimensional poverty ranges from 5.4% in Bogotá to 20.3% in Orinoquía–Amazonía and 18.5% in the Caribbean region [24]. Regional differences in ethnic composition are also pronounced: Indigenous populations are disproportionately represented in Orinoquía–Amazonía (19.2%), the Pacific region (14.2%), and the Atlantic region (10.0%), while Afro-descendant populations are particularly concentrated in the Pacific region (28.5%) [22].We used cross-sectional data from the 2015 Colombian demography and health survey (DHS) [22].

Data were collected in a two-step sampling process; first by geographical area and residence type, then a random household selection. All women who were ever partnered were eligible for the IPV module. Details on the sampling procedure can be found elsewhere [22]. The original DHS dataset contained 38,718 women. Our analytical sample included ever-partnered women aged 15 to 49 years old who were not pregnant, infecund, sterilised, or with a sterilised partner (*n* = 13,896). Women who had been sterilised or whose partners had been sterilised were excluded because sterilisation is intended to be permanent and therefore substantially limits the possibility of changing contraceptive methods in response to recent IPV. Moreover, because the DHS does not report the timing of sterilisation, it was not possible to determine whether sterilisation occurred before or after the IPV exposure assessed in the study. Women who used contraceptive methods that did not fall into the main categories (contraceptive patch, vaginal ring, foam or jelly) were excluded from the analysis (*n* = 18) to keep the contraceptive categories clear (Appendix A).

The outcome variable was current use of reversible contraception, grouped pragmatically according to their characteristics as follows: no contraception, traditional/fertility awareness method (FAM), IUDs/implants, oral/injectable contraceptives, and external condoms. Traditional methods include periodic and prolonged abstinence, withdrawal, and folkloric methods. FAMs, including Lactational Amenorrhoea (LAM) and the Standard Days Method (SDM), were combined with traditional methods due to small sample sizes and their similar characteristics. IUDs and implants were grouped together because these highly effective methods do not require regular healthcare visits once administered and are less susceptible to human error or interference [25]. Oral and injectable method methods, including OCPs, monthly, and three-monthly injections, were grouped together due to their need for frequent replenishment, user dependence, and susceptibility to partner interference [9] External condoms, also known as male condoms, were categorised separately because require partner cooperation and their use is more susceptible to negotiation challenges and may result in less consistent use [9].

Women’s exposure to physical, sexual, emotional IPV, or controlling behaviour in the past 12 months was measured following the WHO definitions [1]. Only IPV in the past year was examined, to better reflect a potential link to current contraception use. Five dichotomous variables were created, capturing whether women had experienced each form of IPV as well as any form of IPV (i.e., if answered ‘yes’ to at least one item).

Physical IPV was defined as any of the following actions: pushing, shaking, throwing something at her, slapping, punching, kicking, dragging, strangling, burning, threatening, or attacking the woman with a knife, gun, or other weapon. Sexual IPV was measured as being forced into unwanted sex. Emotional IPV was captured as being insulted or made feel bad, or when he threatened to leave her or take away the children. Controlling behaviour was defined as whether the partner was jealous if she talked with other men, accused her of unfaithfulness, did not permit her to meet female friends, tried to limit her contact with family, insisted on knowing where she was, did not trust her with money, ignored her or did not request her opinion for family or gatherings or on important family matters.

Statistical analyses were conducted in Stata version 17 using svy command. Data were weighted according to the DHS guidelines [26]. No missing data were present. Significant bivariate associations were assessed using Chi-Squared for categorical variables and Kruskall-Wallis H tests for continuous variables. Multinomial logistic regression estimated adjusted relative risk ratios (aRRR) and 95% confidence intervals (CI) showing the association between IPV exposure in the past 12 months and their current use of different forms of contraception, with non-use as the reference category.

Variables that have been found to be associated with contraceptive use and IPV were included as potential confounders. Potential confounders included the respondent’s age (in 5-year groups), type of place of residence (urban/rural), current union status (married/non-marital/no longer partnered (grouping those widowed/divorced/separated)), parity (number of live or still births), educational level (primary/secondary/higher), whether the respondent was employed, and whether the respondent belonged to a racialised group (including Indigenous, Gypsy, Raizal, Palenguero, Black, Mulato, Afro-Colombian, or Afro-descendant). Region indicated the geographical region of residence of the respondent, based on Colombia’s six-region classification. The DHS household wealth index, measured in quintiles (poorest, poorer, middle, richer, richest), was used to determine the respondent’s relative economic status. A detailed description of the index construction can be found in the DHS manual [26]. Desire for children reflected the respondent’s need for contraception and was categorised into wanting to have another child, undecided or wanting no more.

Multicollinearity was checked via variable inflation factor (VIF), no multicollinearity was found (overall model VIF 1.39, range 1.06 to 1.93 for individual variables). A *p*-value of < 0.05 was considered to be statistically significant.

### Ethical considerations

This study used data collected through the 2015 Colombian DHS, which was fully anonymised and publicly available upon [22]). The procedures for standard DHS surveys have been approved by the International Coaching Federations’ Institutional Review Board. Participants were read an informed consent statement out loud and those who accepted it were able to participate in the survey. Prior to starting the module about IPV, the informed consent was restated and verified. Interviewers ensured privacy and the IPV module was paused or terminated if full privacy could not be guaranteed. When needed, interviewers could provide information and referrals to social and medical services [22]. This study adheres to the STROBE statement for reporting observational research (Appendix B).

### Role of funding source

This study received no funding.

## Results

Table 1 shows the weighted descriptive statistics of the sample. Unweighted descriptive statistics are shown in Appendix C. The overall contraceptive prevalence was 71·4% (Table 1). The two most common methods used were oral and injectable methods (35·3%) and IUD/implants (18·7%). No respondent reported using prolonged abstinence, SDM, or emergency contraception. Appendix D shows a detailed description of the types of contraception used. Around half of the sampled women (51·9%) reported experiencing at least any form of IPV in the past 12 months. Controlling behaviour was the most common IPV type reported (49·1%), followed by emotional IPV (21·1%), physical IPV (17·4%) and sexual IPV (3·3%).

**Table 1:** IPV exposure and socio-demographic characteristics of the study sample stratified by current method of contraception used (weighted column percentages, and p-value shown)

Characteristics	Contraception non-use	Traditional and FAM	IUD and implant	Oral and injectables	External condoms	Total	
Any form of IPV in the past 12 months	55·9%	47·4%	55·5%	49·3%	46·1%	51·9%	< 0·001
Physical IPV in the past 12 months	19·6%	14·3%	19·9%	16·0%	13·5%	17·4%	0·00
Sexual IPV in the past 12 months	3·5%	3·2%	4·3%	2·8%	2·4%	3·3%	0·053
Emotional IPV in the past 12 months	22·1%	20·2%	26·0%	18·0%	17·5%	21·1%	< 0·001
Controlling behaviour in the past 12 months	53·4%	44·7%	51·6%	46·6%	44·5%	49·1%	< 0·001
Age							
15 to 19 years old	5·5%	3·7%	6·0%	7·6%	2·9%	6·0%	< 0·001
20 to 24 years old	14·4%	9·6%	21·8%	24·3%	14·3%	18·9%	
25 to 29 years old	19·5%	16·4%	26·0%	26·4%	18·8%	22·8%	
30 to 34 years old	15·7%	18·3%	17·8%	19·2%	21·6%	18·1%	
35 to 39 years old	14·1%	18·2%	13·6%	12·4%	18·2%	14·1%	
40 to 44 years old	15·0%	17·1%	7·5%	7·0%	14·6%	10·9%	
45 to 49 years old	15·8%	16·7%	7·3%	3·1%	9·6%	9·2%	
Urban residence	78·3%	73·1%	78·9%	72·0%	80·3%	76·0%	< 0·001
Current union status							
Married	13·7%	39·2%	20·9%	21·2%	35·1%	21·8%	< 0·001
Non-marital union	42·9%	51·8%	55·4%	63·4%	49·1%	53·8%	
No longer together with partner	43·4%	9·0%	23·7%	15·4%	15·8%	24·4%	
Parity (median (IQR))	1·0 (1·0)	2·0 (2·0)	1·0 (1·0)	1·0 (1·0)	2·0 (2·0)	1·0 (1·0)	< 0·001
Educational level							
Primary or lower education	22·1%	22·8%	16·0%	18·8%	18·7%	19·5%	< 0·001
Secondary education	41·8%	39·7%	47·8%	48·4%	41·5%	45·1%	
Higher education	36·1%	37·5%	36·2%	32·8%	39·8%	35·4%	
Employed	64·2%	63·8%	66·5%	57·7%	62·7%	62·2%	< 0·001
Racialised ethnic group	16·8%	16·1%	13·2%	14·2%	13·4%	14·8%	0·02
Region							
Atlantic	30·6%	20·6%	13·6%	19·2%	21·7%	21·8%	< 0·001
Orient	15·1%	21·6%	19·4%	15·7%	22·1%	17·3%	
Central	20·9%	22·8%	19·6%	28·4%	20·2%	23·4%	
Pacific	14·9%	18·2%	16·0%	17·8%	14·9%	16·4%	
Bogotá	15·6%	14·5%	28·2%	15·8%	17·9%	18·1%	
Orinoquia/ Amazonia	2·9%	2·3%	3·2%	3·1%	3·2%	3·0%	
Household wealth							
Poorest	19·7%	22·7%	18·0%	24·4%	15·5%	20·9%	< 0·001
Poorer	23·1%	18·9%	20·0%	22·8%	18·0%	21·6%	
Middle	21·8%	17·7%	21·4%	22·4%	24·4%	21·9%	
Richer	19·3%	17·7%	21·7%	15·0%	24·6%	18·6%	
Richest	16·1%	23·0%	18·9%	15·4%	17·5%	17·0%	
Desire for children							
Have another	54·7%	37·4%	43·9%	49·4%	36·5%	47·7%	< 0·001
Undecided	2·5%	3·5%	4·4%	4·2%	2·4%	3·5%	
No more	42·8%	59·1%	51·7%	46·4%	61·1%	48·8%	
Total	28·6%	7·9%	18·7%	35·3%	9·5%	100%	

Percentages are weighted; unweighted sample size: *n* = 13,896

*Abbreviations*: *FAM* fertility awareness method, *IPV* intimate partner violence, *IUD* intrauterine device, *IQR* interquartile range

Most women were young with 47·7% of the sample aged 20–29 years or less. Seven in ten women lived in an urban setting and 53·8% lived with a partner without being married (Table 1). In addition, educational levels were generally high with 45·1% reporting secondary education and 35·4% tertiary education. Around one in seven (14·8%) of the sample reported belonging to a racialised ethnic group. Almost half of the sample resided in the Central (23·4%) or Atlantic (21·8%) regions. Almost half of the sample reported desiring no more children, and the median parity was 1 child per woman (interquartile range (IQR) = 1).

Bivariate analysis revealed significant differences in IPV exposure across users of different contraceptive methods (Table 1). Women who used IUDs/implants or did not use any contraceptive method reported higher prevalence rates of all forms of IPV compared to users of other contraceptive methods. Notably, 53·4% of non-users reported experiencing controlling behaviour—higher than among any group of contraceptive users. Additionally, women using IUDs/implants reported elevated risks of sexual IPV (4·3%) and emotional IPV (26·0%) compared to both users of other contraceptive methods and non-users. However, there were no statistically significant differences in contraceptive methods in relation to sexual IPV.

Contraceptive use patterns also varied significantly across sociodemographic groups (Table 1). For instance, the use of IUDs/implants (26·0%) and oral/injectable contraceptives (26·4%) was most common among women aged 25–29 years. In contrast, non-use and reliance on traditional or FAMs was more common among older women. Use of IUDs/implants (78·9%) and external condoms (80·3%) was more prevalent in urban areas. Additionally, residents in the Atlantic region (30·6%) and those belonging to a racialised ethnic group (16·8%) reported the highest rates of non-contraceptive use.

Oral and injectable contraceptive use was more common among women in non-marital unions (63·4%). A substantial proportion of non-users (22·1%) or respondents relying on traditional or FAMs (22·8%) reported having only primary education or less. Employment was more prevalent among women using IUDs/implants, with 66·5% reporting that they were working. Lastly, users of traditional or FAMs and external condoms generally had a higher parity, with a median of 2 children (IQR = 1).

Table 2 presents the results of the multivariable analyses estimating adjusted associations between IPV exposure and current contraceptive method, in reference to non-users and those who did not experience the different forms of IPV. Each line in the table describes a separate multinomial logistic regression. Adjusted associations varied by IPV type and contraceptive method, with some forms being linked to higher use of specific methods and other forms to lower use.

**Table 2:** Adjusted associations between exposure to different forms of IPV in the last 12 months and current use of different types of contraceptive methods (weighted)

	Traditional and FAM	IUD and implant	Oral and injectables	External condom
	aRRR (95% CI)	aRRR (95% CI)	aRRR (95% CI)	aRRR (95% CI)
Any form of IPV	0·87 (0·75 − 1·01)	0·98 (0·88 − 1·09)	0·74 (0·67 − 0·81)	0·76 (0·66 − 0·87)
Physical IPV	1·12 (0·91 − 1·37)	1·16 (1·01–1·33)	0·91 (0·80 − 1·03)	0·87 (0·72 − 1·06)
Sexual IPV	1·58 (1·05 − 2·38)	1·73 (1·30 − 2·29)	1·18 (0·90 − 1·55)	1·05 (0·69 − 1·59)
Emotional IPV	1·23 (1·02 − 1·47)	1·43 (1·26 − 1·63)	0·96 (0·85 − 1·08)	0·94 (0·79 − 1·12)
Controlling behaviour	0·84 (0·73 − 0·97)	0·90 (0·81 − 1·01)	0·72 (0·65 − 0·79)	0·77 (0·68 − 0·89)

Each line represents a multinominal logistic regression. Data were weighted; unweighted sample size: *n* = 13,896. Adjusted for age, residence, current union status, parity, educational status, employment status, racialised ethnic group, region, household wealth and desire for children. Reference category: contraceptive non-use

*Abbreviations*: *aRRR* adjusted relative risk ratio, *CI* confidence interval, *FAM* fertility awareness method, *IPV* intimate partner violence, *IUD* intrauterine device

Compared to non-users, those exposed to any form of IPV in the past 12 months had a significantly lower relative risk of using oral and injectable methods (aRRR = 0·74, 95% CI [0·67–0·81]) or external condoms (aRRR = 0·76, 95% CI [0·66–0·87]). Additionally, women who experienced controlling behaviour had lower relative risk of using OCPs and injectables (aRRR = 0·72, 95% CI [0·65–0·79]), external condoms (aRRR = 0·77, 95% CI [0·68–0·89]), or traditional methods and FAMs (aRRR = 0·84, 95% CI [0·73–0·97]).

Conversely, physical, sexual, and emotional IPV in the last 12 months were associated with a higher relative risk of using some contraceptive methods. Women who experienced physical (aRRR = 1·16, 95% CI [1·01–1·33]), sexual (aRRR = 1·73, 95% CI [1·30–2·29]), or emotional IPV (aRRR = 1·43, 95% CI [1·26–1·63]) had a higher relative risk of using IUDs or implants. Similarly, sexual (aRRR = 1·58, 95% CI [1·05–2·38]) and emotional IPV (aRRR = 1·23, 95% CI [1·02–1·47]) were associated with a higher relative risk of using traditional methods or FAMs.

## Discussion

Although IPV has been linked with contraception use in different contexts, little is known about this association in Colombia, a country with both high IPV prevalence and widespread contraception use. In our study, 71% of the women used reversible contraception methods, and 1 in 2 women reported to have experienced any form of IPV in the last 12 months. Oral/injectable methods were the most common used methods (35·3%). The prevalences of IPV and contraception use in our study align with previous studies in Colombia and Latin America [5, 16, 19, 27].

Associations between different forms of IPV in the last 12 months and current reversible contraceptive use differed depending on the specific type of IPV and the type of contraceptive used. This may explain differences across previous studies only focusing on overall IPV experiences or use of any contraceptive method, as use of specific methods may be more influenced by experiencing specific forms of IPV. After adjusting for confounding factors, exposure to any form of IPV was associated with a lower relative risk of using external condoms or oral/injectables, confirming our hypothesis and aligning with findings from India [28] and the United States [11]. Reduced external condom use might reflect difficulties in contraceptive negotiation in contexts of fear, coercion, or a broader power imbalance within the relationship [9, 10].

Reduced relative risk of using oral contraceptives with IPV has been reported previously in the United States [9], and IPV exposure has been also linked to inconsistent oral contraception use, but little is known about the relation between injectables and IPV exposure. Despite being female-controlled, both methods are user dependent and require regular adherence and replenishment, making them vulnerable to partner interference. A greater visibility and the need for routine action may explain why women use these methods less when experiencing IPV [14]. Additionally, IPV-affected women are at higher risk of depression and other mental health issues [9, 29], which could affect their ability to adhere to methods such as injections and OCPs. While exposure to any form of IPV and controlling behaviour was significantly associated with lower oral/injectable contraceptive use, no significant associations were found when we analysed physical, sexual, or emotional IPV exposures separately.

Exposure to physical, sexual, and emotional IPV in the previous 12 months was significantly associated with a higher relative risk of using IUD/implants, supporting our hypothesis. This aligns with studies from the United States [11] showing increased IUD/implant use among women aged 18–19 years exposed to physical IPV, and from Uganda [30] reporting that women exposed to physical, sexual or emotional IPV were more likely to use long-acting reversible contraception (LARC), including IUDs and implants. One possible explanation for this positive association is that IUDs and implants are long-acting and require no daily maintenance and therefore reduce the risk for partner interference once administered and offering a strategic advantage for women in controlling their fertility in the context of IPV. Another explanation is that IUDs and implants can be used covertly. Previous studies have shown that women experiencing IPV are more inclined to use their methods without disclosing it to their partners [10, 14, 15].

Surprisingly, our data also showed that exposure to sexual or emotional IPV in the last 12 months were associated with a higher relative risk of using traditional/FAMs, aligning with results from a multi-country Sub-Saharan African study [13]. Women might turn to traditional/FAM in an attempt to maintain control over their fertility when access to modern contraceptives is restricted or when they fear violence from raising the topic of contraception with their partners. Methods such as withdrawal are also male-dependent and may function as a way to maintain reproductive control over female partners [31].

Our data also showed that controlling behaviour was consistently associated with lower use of oral/injectables, external condoms, and traditional/FAM methods. Controlling behaviour is the most common form of IPV in Colombia and many other settings [32], and has been shown to harm women’s sexual health, due to higher risks of contracting HIV [33], and unintended pregnancies [28] among others.Controlling behaviour can impair women’s contraceptive use and reproductive autonomy through different pathways. For example, by restricting access to health services [34] which can interfere with timely, effective, and consistent access and use of contraceptive methods.

### Strengths and limitations

A notable strength of this study is the differentiation between different contraceptive methods and between various forms of IPV, including controlling behaviour, which is often overlooked in similar research. By distinguishing among contraceptive methods, we provide a more nuanced understanding of how specific types of IPV influence different contraceptive choices, since some methods may be more susceptible to partner interference and others may be adopted as a way of regaining reproductive autonomy when exposed to violence. Furthermore, the use of a large, nationally representative DHS dataset enhances the generalisability of the findings to the Colombian population and potentially to other Latin American countries with similar social and cultural contexts.

However, several limitations should be noted. The cross-sectional design of the survey restricts our ability to draw causal conclusions. In addition, the DHS did not collect information on whether women were using their preferred contraceptive method, or whether non-users wanted to use contraception but were unable to, limiting our understanding of women’s autonomy in contraceptive decision-making. The survey also did not assess whether contraceptives were used consistently or effectively.

Furthermore, the categorisation of current contraceptive use in the DHS is mutually exclusive, potentially leading to underreporting of use of multiple methods concurrently, such as condom use for STI protection in combination with another method. IPV may be underreported due to stigma, fear, or social desirability bias, either because respondents do not answer the questions honestly or because they were excluded from the IPV module due to privacy concerns. The measurement of sexual IPV was limited to being forced into unwanted sex, potentially missing other forms of sexual violence and underestimating its actual prevalence. There may be temporal misalignment between IPV exposure in the past 12 months and current contraceptive use, particularly for long-acting methods initiated before the reported IPV. Additionally, hormonal methods may have been used for non-contraceptive medical reasons, which the survey could not distinguish. Finally, the data were collected before the COVID-19 pandemic, which might have affected both IPV prevalence and access to reproductive health services. As such, the situation regarding IPV and contraception in Colombia may have changed since the time of data collection.

## Conclusions

Our study suggests that Colombian women exposed to different forms of IPV adapt their contraceptive choices in ways that reflect both partner interference and strategies to preserve autonomy. Compared to non-users, those exposed to IPV were less likely to use oral/injectable methods and condoms, particularly under controlling behaviour, underscoring vulnerability to reproductive coercion. In contrast, physical, sexual, and emotional IPV were associated with greater use of IUDs, implants, and in some cases traditional methods, pointing to covert or risk-minimizing strategies in unsafe relationships. Policies to strengthen reproductive autonomy should include routine IPV screening, including controlling behaviours, within reproductive health services, alongside provider training to recommend safe contraceptive options and ensure clear referral pathways to IPV support. Expanding access to IUDs and implants is critical, given their resilience to partner interference, and should be complemented by gender-transformative community interventions targeting unequal norms that sustain IPV and reproductive control.

## Supplementary Information

Supplementary Material 1.

## Data Availability

The datasets used and/or analysed during the current study are available from the corresponding author on reasonable request.

## Abbreviations

aRRR: Adjusted Relative Risk Ratios
CI: Confidence Intervals
DHS: Demography and Health Survey
IPV: Intimate Partner Violence
IUDs: Intrauterine Devices
FAM: Traditional/Fertility Awareness Methods
LARC: Long-acting Reversible Contraception
LAM: Lactational Amenorrhoea
SDGs: Sustainable Development Goals
STIs: Sexually Transmitted Infections
SDM: Standard Days Method
VIF: Variable Inflation Factor
WHO: World Health Organization
