# Supplementary material for: Contraceptive method choice by type of intimate partner violence: findings from the 2015 Colombian demographic and health survey
Source: Reprod Health. 2026 Oct 24;23:193. doi: 10.1186/s12978-026-02478-8 (PMC13695270; doi:10.1186/s12978-026-02478-8)
Supplement: Supplementary file 1 [file 12978_2026_2478_MOESM1_ESM.docx]

**Annex A. Formation of the study sample with the inclusion and exclusion criteria**

**Abbreviations**: FAM = fertility awareness method, IPV = intimate partner violence, IUD = intrauterine device, SD = standard deviation

**Annex B: STrengthining the Reporting of OBservational studies in Epidemiology (STROBE) checklist for cross-sectional studies.**

|  | **Item Number** | **Recommendation** | **Reported on page number** |
| --- | --- | --- | --- |
| **Title and abstract** | 1 | (*a*) Indicate the study’s design with a commonly used term in the title or the abstract | See page 1 |
|  |  | (*b*) Provide in the abstract an informative and balanced summary of what was done and what was found | See page 3-4 |
| **Introduction** | | |  |
| Background/rationale | 2 | Explain the scientific background and rationale for the investigation being reported | See page 7-9 |
| Objectives | 3 | State specific objectives, including any prespecified hypotheses | See page 9 |
| **Methods** | | |  |
| Study design | 4 | Present key elements of study design early in the paper | See page 9 |
| Setting | 5 | Describe the setting, locations, and relevant dates, including periods of recruitment, exposure, follow-up, and data collection | See page 9 |
| Participants | 6 | (*a*) Give the eligibility criteria, and the sources and methods of selection of participants | See page 9-10 |
| Variables | 7 | Clearly define all outcomes, exposures, predictors, potential confounders, and effect modifiers. Give diagnostic criteria, if applicable | See page 10-12 |
| Data sources/ measurement | 8* | For each variable of interest, give sources of data and details of methods of assessment (measurement). Describe comparability of assessment methods if there is more than one group | See page 9-10 |
| Bias | 9 | Describe any efforts to address potential sources of bias | See page 18-20 |
| Study size | 10 | Explain how the study size was arrived at | See page 9-10 |
| Quantitative variables | 11 | Explain how quantitative variables were handled in the analyses. If applicable, describe which groupings were chosen and why | See page 11-12 |
| Statistical methods | 12 | (*a*) Describe all statistical methods, including those used to control for confounding | See page 11-12 |
|  |  | (*b*) Describe any methods used to examine subgroups and interactions | See page 11-12 |
|  |  | (*c*) Explain how missing data were addressed | See page 11 |
|  |  | (*d*) If applicable, describe analytical methods taking account of sampling strategy | Not applicable |
|  |  | (*e*) Describe any sensitivity analyses | Not applicable |
| **Results** | | |  |
| Participants | 13* | (a) Report numbers of individuals at each stage of study—eg numbers potentially eligible, examined for eligibility, confirmed eligible, included in the study, completing follow-up, and analysed | See page 9-10 |
|  |  | (b) Give reasons for non-participation at each stage | See page 10 |
|  |  | (c) Consider use of a flow diagram | See page 32 |
| Descriptive data | 14* | (a) Give characteristics of study participants (eg demographic, clinical, social) and information on exposures and potential confounders | See page 13, 15, 16 |
|  |  | (b) Indicate number of participants with missing data for each variable of interest | See page 11 |
| Outcome data | 15* | Report numbers of outcome events or summary measures | See page 13-16 |
| Main results | 16 | (*a*) Give unadjusted estimates and, if applicable, confounder-adjusted estimates and their precision (eg, 95% confidence interval). Make clear which confounders were adjusted for and why they were included | See page 17-18 |
|  |  | (*b*) Report category boundaries when continuous variables were categorized | Not applicable |
|  |  | (*c*) If relevant, consider translating estimates of relative risk into absolute risk for a meaningful time period | Not applicable |
| Other analyses | 17 | Report other analyses done—eg analyses of subgroups and interactions, and sensitivity analyses | Not applicable |
| **Discussion** | | |  |
| Key results | 18 | Summarise key results with reference to study objectives | See page 18 |
| Limitations | 19 | Discuss limitations of the study, taking into account sources of potential bias or imprecision. Discuss both direction and magnitude of any potential bias | See page 20-21 |
| Interpretation | 20 | Give a cautious overall interpretation of results considering objectives, limitations, multiplicity of analyses, results from similar studies, and other relevant evidence | See page 18-21 |
| Generalisability | 21 | Discuss the generalisability (external validity) of the study results | See page 20-21 |
| **Other information** | | |  |
| Funding | 22 | Give the source of funding and the role of the funders for the present study and, if applicable, for the original study on which the present article is based | See page 2 |

*Give information separately for exposed and unexposed groups.

**Note:** An Explanation and Elaboration article discusses each checklist item and gives methodological background and published examples of transparent reporting. The STROBE checklist is best used in conjunction with this article (freely available on the Web sites of PLoS Medicine at http://www.plosmedicine.org/, Annals of Internal Medicine at http://www.annals.org/, and Epidemiology at http://www.epidem.com/). Information on the STROBE Initiative is available at www.strobe-statement.org.

**Annex C. IPV exposure and sociodemographic characteristics of the study sample by current contraceptive method, 2015 Colombian Demographic and Health Survey (unweighted percentages).**

| **Characteristics** | | **Contraception non-use** | **Traditional and FAM** | **IUD/implant** | **Oral and injectables** | **External condoms** | **Total** |  |
| --- | --- | --- | --- | --- | --- | --- | --- | --- |
|  | | Frequency (%) / median (IQR) | | | | | | *p-*value |
| Any form of IPV in the past 12 months | | 2270 (54.2%) | 538 (51.2%) | 1386 (58.2%) | 2574 (52.3%) | 643 (47.7%) | 7411 (53.3%) | < 0.001 |
| Physical IPV in the past 12 months | | 882 (21.0%) | 185 (17.6%) | 535 (22.5%) | 873 (17.7%) | 197 (14.6%) | 2672 (19.2%) | < 0.001 |
| Sexual IPV in the past 12 months | | 172 (4.1%) | 37 (3.5%) | 118 (5.0%) | 149 (3.0%) | 40 (2.9%) | 516 (3.7%) | 0.000 |
| Emotional IPV in the past 12 months | | 991 (23.6%) | 239 (22.7%) | 638 (26.8%) | 1065 (21.6%) | 249 (18.6%) | 3182 (22.9%) | < 0.001 |
| Controlling behaviour in the past 12 months | | 2130 (50.8%) | 500 (47.6%) | 1298 (54.5%) | 2401 (48.8%) | 616 (45.7%) | 6945 (50.0%) | < 0.001 |
| Age | |  |  |  |  |  |  | < 0.001 |
|  | 15 to 19 years old | 272 (6.5%) | 54 (5.1%) | 186 (7.8%) | 438 (8.9%) | 53 (3.9%) | 1003 (7.2%) |  |
|  | 20 to 24 years old | 612 (14.6%) | 141 (13.4%) | 537 (22.6%) | 1179 (23.9%) | 189 (14.0%) | 2658 (19.1%) |  |
|  | 25 to 29 years old | 741 (17.7%) | 152 (14.5%) | 606 (25.5%) | 1201 (24.4%) | 261 (19.4%) | 2961 (21.3%) |  |
|  | 30 to 34 years old | 715 (17.1%) | 159 (15.1%) | 444 (18.7%) | 932 (18.9%) | 264 (19.6%) | 2514 (18.1%) |  |
|  | 35 to 39 years old | 599 (14.3%) | 192 (18.3%) | 286 (12.0%) | 587 (11.9%) | 263 (19.5%) | 1927 (13.9%) |  |
|  | 40 to 44 years old | 572 (13.6%) | 186 (17.7%) | 190 (8.0%) | 390 (7.9%) | 187 (13.9%) | 1525 (11.0%) |  |
|  | 45 to 49 years old | 681 (16.2%) | 167 (15.9%) | 131 (5.5%) | 201 (4.0%) | 131 (9.7%) | 1311 (9.4%) |  |
| Urban residence | | 3043 (72.6%) | 683 (65.0%) | 1764 (74.1%) | 3362 (68.3%) | 1037 (76.9%) | 9889 (71.2%) | < 0.001 |
| Current union status | |  |  |  |  |  |  | < 0.001 |
|  | Married | 602 (14.4%) | 332 (31.6%) | 436 (18.3%) | 897 (18.2%) | 432 (32.0%) | 2699 (19.4%) |  |
|  | Non-marital union | 1869 (44.6%) | 629 (59.8%) | 1410 (59.2%) | 3299 (67.0%) | 700 (51.9%) | 7907 (56.9%) |  |
|  | No longer together with partner | 1721 (41.1%) | 90 (8.6%) | 534 (22.4%) | 729 (14.8%) | 216 (16.0%) | 3290 (23.7%) |  |
| Parity (median (IQR)) | | 1·0 (2·0) | 2·0 (2·0) | 2·0 (1·0) | 2·0 (1·0) | 2·0 (2·0 | 2·0 (1·0) | < 0.001 |
| Educational level | |  |  |  |  |  |  | < 0.001 |
|  | Primary or lower education | 1118 (26.7%) | 339 (32.3%) | 486 (20.4%) | 1155 (23.5%) | 272 (20.2%) | 3370 (24.3%) |  |
|  | Secondary education | 1778 (42.4%) | 436 (41.5%) | 1200 (50.4%) | 2375 (48.2%) | 574 (42.6%) | 6363 (45.8%) |  |
|  | Higher education | 1296 (30.9%) | 276 (26.3%) | 694 (29.2%) | 1395 (28.3%) | 502 (37.2%) | 4163 (30.0%) |  |
| Employed | | 2657 (63.4%) | 629 (59.8%) | 1476 (62.0%) | 2675 (54.3%) | 838 (62.2%) | 8275 (59.5%) | < 0.001 |
| Racialised ethnic group | | 1165 (27.8%) | 306 (29.1%) | 517 (21.7%) | 1096 (22.3%) | 217 (16.1%) | 3302 (23.8%) | < 0.001 |
| Department | |  |  |  |  |  |  | < 0.001 |
|  | Atlantic | 1370 (32.7%) | 253 (24.1%) | 381 (16.0%) | 1076 (21.8%) | 303 (22.5%) | 3383 (24.3%) |  |
|  | Orient | 467 (11.1%) | 177 (16.8%) | 394 (16.6%) | 648 (13.2%) | 242 (18.0%) | 1928 (13.9%) |  |
|  | Central | 788 (18.8%) | 208 (19.8%) | 487 (20.5%) | 1233 (25.0%) | 279 (20.7%) | 2995 (21.6%) |  |
|  | Pacific | 621 (14.8%) | 209 (19.9%) | 396 (16.6%) | 815 (16.5%) | 198 (14.7%) | 2239 (16.1%) |  |
|  | Bogotá | 197 (4.7%) | 39 (3.7%) | 263 (11.1%) | 243 (4.9%) | 100 (7.4%) | 842 (6.1%) |  |
|  | Orinoquia / Amazonia | 749 (17.9%) | 165 (15.7%) | 459 (19.3%) | 910 (18.5%) | 226 (16.8%) | 2509 (18.1%) |  |
| Household wealth | |  |  |  |  |  |  | < 0.001 |
|  | Poorest | 1226 (29.2%) | 367 (34.9%) | 653 (27.4%) | 1565 (31.8%) | 280 (20.8%) | 4091 (29.4%) |  |
|  | Poorer | 1328 (31.7%) | 284 (27.0%) | 712 (29.9%) | 1540 (31.3%) | 376 (27.9%) | 4240 (30.5%) |  |
|  | Middle | 857 (20.4%) | 181 (17.2%) | 486 (20.4%) | 995 (20.2%) | 307 (22.8%) | 2826 (20.3%) |  |
|  | Richer | 509 (12.1%) | 135 (12.8%) | 319 (13.4%) | 545 (11.1%) | 244 (18.1%) | 1752 (12.6%) |  |
|  | Richest | 272 (6.5%) | 84 (8.0%) | 210 (8.8%) | 280 (5.7%) | 141 (10.5%) | 987 (7.1%) |  |
| Desire for children | |  |  |  |  |  |  | < 0.001 |
|  | Have another | 2158 (51.5%) | 386 (36.7%) | 1066 (44.8%) | 2272 (46.1%) | 503 (37.3%) | 6385 (45.9%) |  |
|  | Undecided | 1919 (45.8%) | 634 (60.3%) | 1214 (51.0%) | 2479 (50.3%) | 800 (59.3%) | 7046 (50.7%) |  |
|  | No more | 115 (2.7%) | 31 (2.9%) | 100 (4.2%) | 174 (3.5%) | 45 (3.3%) | 465 (3.3%) |  |
| **Total** | | **4192** | **1051** | **2380** | **4943** | **1348** | **13,896** |  |

**Abbreviations**: FAM = fertility awareness method, IPV = intimate partner violence, IUD = intrauterine device, IQR = interquartile range

**Appendix D. Current contraceptive method use in the study sample, 2015 Colombian Demographic and Health Survey (weighted percentages).**

| **Type of contraception** | | **%** |
| --- | --- | --- |
| **No contraception** | | **28.6%** |
| **Traditional and fertility awareness method** | | **7.9%** |
|  | Withdrawal | 4.4% |
|  | Periodic abstinence | 3.0% |
|  | Lactational Amenorrhoea (LAM) | 0.2% |
|  | Folkloric methods | 0.3% |
| **IUD and implant** | | **18.7%** |
|  | Intrauterine device (IUD) | 9.0% |
|  | Implants | 9.7% |
| **Oral and injectable** | | **35.3%** |
|  | Oral contraceptive pills (OCPs) | 11.8% |
|  | Monthly injection | 15.6% |
|  | Three months injection | 7.9% |
| **External condom** | | **9.5%** |

**Note**: Percentages are weighted; unweighted sample size: n = 13,896.
